# Assessment of somatic mutations in urine and plasma of Wilms tumor patients

**DOI:** 10.1002/cam4.3236

**Published:** 2020-06-26

**Authors:** Ana Carolina Kerekes Miguez, Bruna D. de Figueiredo Barros, Jorge E. S. de Souza, Cecília Maria L. da Costa, Isabela Werneck Cunha, Paula Nicole Vieira P. Barbosa, Maria Lúcia P. Apezzato, Sandro J. de Souza, Dirce Maria Carraro

**Affiliations:** ^1^ Laboratory of Genomics and Molecular Biology International Research Center/CIPE A. C. Camargo Cancer Center São Paulo Brazil; ^2^ Bioinformatics Multidisciplinary Environment Digital Metropolis Institute Federal University of Rio Grande do Norte Natal Brazil; ^3^ Pediatrics Department A. C. Camargo Cancer Center São Paulo Brazil; ^4^ Anatomic Pathology Department A. C. Camargo Cancer Center São Paulo Brazil; ^5^ Imaging Department A. C. Camargo Cancer Center São Paulo Brazil; ^6^ Pediatric Surgery Department A. C. Camargo Cancer Center São Paulo Brazil; ^7^ Brain Institute Federal University of Rio Grande do Norte Natal Brazil; ^8^ National Institute of Science and Technology in Oncogenomics and Therapeutic Innovation (INCITO) São Paulo Brazil

**Keywords:** cancer genetics, liquid biopsy, pediatric cancer, Wilms tumor

## Abstract

Tumor DNA has been detected in body fluids of cancer patients. Somatic tumor mutations are being used as biomarkers in body fluids to monitor chemotherapy response as a minimally invasive tool. In this study, we evaluated the potential of tracking somatic mutations in free DNA of plasma and urine collected from Wilms tumor (WT) patients for monitoring treatment response. Wilms tumor is a pediatric renal tumor resulting from cell differentiation errors during nephrogenesis. Its mutational repertoire is not completely defined. Thus, for identifying somatic mutations from tumor tissue DNA, we screened matched tumor/leukocyte DNAs using either a panel containing 16 WT‐associated genes or whole‐exome sequencing (WES). The identified somatic tumor mutations were tracked in urine and plasma DNA collected before, during and after treatment. At least one somatic mutation was identified in five out of six WT tissue samples analyzed. Somatic mutations were detected in body fluids before treatment in all five patients (three patients in urine, three in plasma, and one in both body fluids). In all patients, a decrease of the variant allele fraction of somatic mutations was observed in body fluids during neoadjuvant chemotherapy. Interestingly, the persistence of somatic mutations in body fluids was in accordance with clinical parameters. For one patient who progressed to death, it persisted in high levels in serial body fluid samples during treatment. For three patients without disease progression, somatic mutations were not consistently detected in samples throughout monitoring. For one patient with bilateral disease, a somatic mutation was detected at low levels with no support of clinical manifestation. Our results demonstrated the potential of tracking somatic mutations in urine and plasma DNA as a minimally invasive tool for monitoring WT patients. Additional investigation is needed to check the clinical value of insistent somatic mutations in body fluids.

## Graphical abstract

Here we presented the use of urine and plasma samples of pediatric patients diagnosed with Wilms tumor (WT) as a minimally invasive tool for monitoring the treatment response. We were able to detect tumor‐specific somatic mutations in free DNA from both body fluids, observing a correlation with the clinical aspects of these patients.

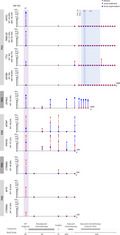

## 1 INTRODUCTION

Wilms tumor (WT) is a tumor of embryonic origin that occurs due to errors in the differentiation process in primitive cells during the early stages of nephrogenesis.^1^, ^2^ Histologically, it may contain tissue components of three different morphologies: mesenchymal/stromal, epithelial, and blastemal, recapitulating the different stages of kidney development.

The majority of WT cases are sporadic, resulting from somatic mutations, which usually are restricted to tumor tissue.^3^ So far, few tumor causing mutations have been reported in a few genes, frequently associated with molecular pathways involved in cell differentiation (such as *CTNNB1*, *APC*, *WTX*, and *TP53*, which are related to the Wnt signaling pathway^4^, ^5^, ^6^, ^7^; *WT1*, *MYCN*, and *SIX1/2*, which are critical for early renal development^5^, ^8^, ^9^, ^10^, ^11^) or posttranscriptional regulation (like the microRNA processor genes *DROSHA*, *DICER1*, *XPO5*, *TARBP2*, and *DGCR8* ^9^, ^10^, ^11^, ^12^, ^13^). However, about 50% of cases present mutations in other genes not yet associated with WT,^12^ demonstrating a great genetic heterogeneity in this neoplasia and leaving a gap in the understanding of the different types of biological processes and pathways that might be operating in WT tumorigenesis.

The identification of tumor‐specific genetic alterations has additional value for precisely tracking tumor DNA in body fluids of WT patients. The detection of somatic mutations in plasma has been recognized as being clearly representative of the tumor genome for different types of solid tumors,^14^, ^15^ displaying a high correlation between mutation allele fractions and disease progression.^14^, ^16^ Thus, screening of somatic mutations in body fluids, a noninvasive approach, is a promising tool for patient stratification during therapy and for early monitoring of recurrence. Besides plasma, urine also has been explored as a completely noninvasive liquid biopsy alternative,^17^, ^18^, ^19^ especially for patients presenting urogenital cancers.^20^, ^21^, ^22^, ^23^, ^24^, ^25^, ^26^, ^27^, ^28^

For WT patients, a few studies have analyzed liquid biopsy samples. The detection of tumor‐specific *TP53* mutations has been reported in plasma and serum from patients with diffuse anaplastic WT (DAWT),^29^ and tumor‐specific copy number alterations and single nucleotide variants have been observed in plasma from WT patients before nephrectomy.^30^ However, liquid biopsy using plasma and urine has not been explored yet as a potential approach for monitoring response throughout treatment.

Thus, in this study, we used a panel of WT‐associated genes, previously published by us,^12^ to identify somatic mutations in WT as well as whole‐exome sequencing (WES) for the tumors negative for a somatic mutation in the genes included in the panel. Somatic mutations of each tumor were screened using deep amplicon sequencing of plasma DNA and of sediment and supernatant DNA of urine samples before, during, and after the treatment, in a personalized format, suggesting the presence of tumor DNA. The presence or absence, as well as the level of somatic mutations in serial body fluids samples were then correlated with clinical aspects of each patient.

## 2 MATERIALS AND METHODS

### 2.1 Biological sample collection

All patients from the AC Camargo Cancer Center pediatric oncology department diagnosed with WT from 2014 to 2016 were invited to participate in this study. The legally responsible relative of each patient signed an informed consent, and biological samples were obtained in accordance with the AC Camargo Cancer Center Ethics Committee.

Wilms tumor tissue sample from each patient was collected during surgery and subjected to histological analysis by a pathologist to assess the suitability of the malignant tumor tissue. Samples were manually dissected, and areas containing non‐neoplastic tissues, fibrosis, or other contaminants were removed by macrodissection when necessary. All WT samples used in this study submitted to DNA isolation displayed at least 70% of tumor cells.

**FIGURE 1:**
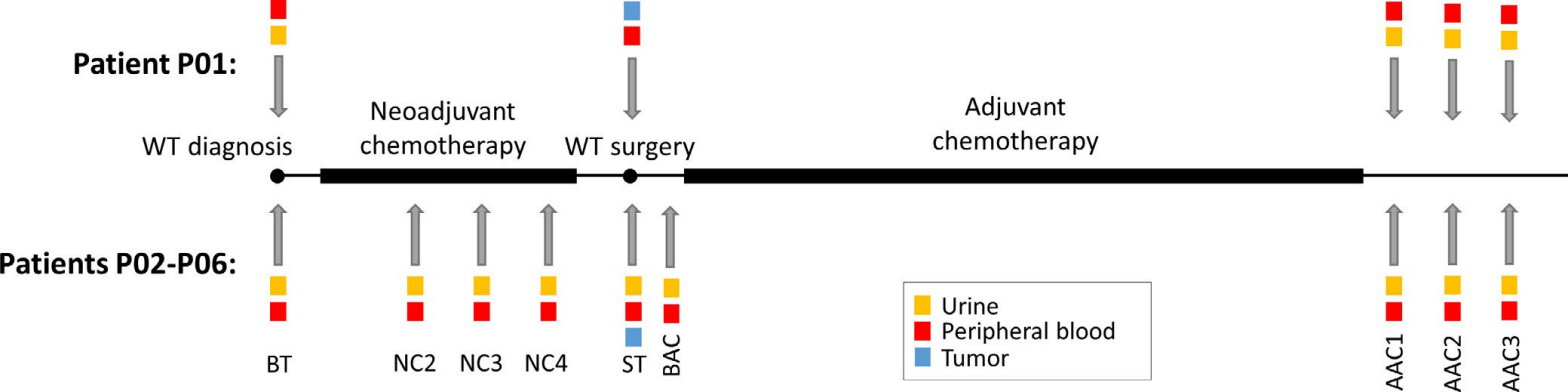
Collection of tumor tissue and body fluids before, during, and after treatment. BT, before treatment; NC2‐NC4, neoadjuvant chemotherapy at weeks 2‐4; ST, time of surgery; BAC, before adjuvant chemotherapy; AAC1‐AAC3, after adjuvant chemotherapy

Blood and urine samples were collected at different time points of treatment, as indicated in Figure 1. Patient P01 had biological samples collected before and after treatment (neo‐ and adjuvant chemotherapy) for primary tumor and during the metastasis treatment. Patients P02‐P06 had samples collected before, during, and after treatment (neo‐ and adjuvant chemotherapy) for primary tumor.

### 2.2 DNA isolation

Genomic DNA isolation from freshly frozen tumor tissue was performed using the phenol:chloroform method. Peripheral blood for leukocyte DNA analysis was collected in EDTA tubes, and DNA extraction was performed using QIASymphony SP equipment (QIAGEN) using the QIASymphony DNA Midi Kit (QIAGEN), according to the Standard Operating Procedure (SOP) of the AC Camargo Cancer Center Biobank.

Regarding body fluids, urine and blood samples were processed within 2 hours after collection. For plasma samples, 5 mL of peripheral blood was collected in Vacutainer Plasma Preparation Tubes (PPT) (BD, USA) and followed to centrifugation of 3,000 rpm during 15 minutes in ambient temperature. The plasma was carefully transferred into a 15‐mL FALCON tube and submitted again to centrifugation of 3,000 rpm for 15 minutes. Plasma samples were stored at −80°C. DNA isolation from plasma was performed using a QIAamp DNA Blood Midi Kit (QIAGEN). For urine samples, DNA isolated from the two components were analyzed, sediment and supernatant. A total of 50 mL of urine was collected in FALCON tubes and sediment and supernatant components were separated by centrifugation of 3,000 rcf for 10 minutes in room temperature. Urine supernatant was transferred to a 50‐mL FALCON tube and urine sediment was transferred to a 1.5‐mL Eppendorf tube (along with a residual supernatant volume of approximately 100 μL). Urine supernatants and sediments were stored at −80°C. DNA isolation was performed on a QIASymphony SP equipment (QIAGEN) using the QIASymphony DNA Midi Kit (QIAGEN) with adaptations for urine sediment samples: 20 μL of proteinase K (20 mg/mL; QIAGEN, Germany) and 20 μL of β‐mercaptoethanol (Sigma‐Aldrich, USA), and 1000 μL of ATL lysis buffer (QIAGEN) were used for optimization of cell lysis. This solution was homogenized by vortex for 1 minute and held at 900 rpm and 56°C for 2 hours for initial cell lysis. For urine supernatant, the QIASymphony DNA Midi Kit (QIAGEN) was used according to the manufacturer's instructions.

DNA quantification was obtained using NanoDrop ND 1000 (Thermo Fisher Scientific). The amount of DNA recovered from body fluids is available in Table S1. The analysis of fragment size profile of DNA from plasma samples was performed using the 2100 Bioanalyzer instrument (Agilent) with the Bioanalyzer High Sensitivity DNA Kit (Agilent). We were able to evaluate 70% of the samples, which had enough DNA for the analysis, and in all of them we observe a peak in the interval of 140‐170 bp reflecting the presence of cell free DNA and no detectable contamination of genomic DNA from lysis of blood cells.

### 2.3 Target parallel sequencing of the WT‐gene panel and WES for detecting somatic mutations in WT tissue

Tumor tissue and leukocyte DNAs were sequenced for detecting tumor somatic mutations using a WT‐gene panel from a previous study^12^ and/or whole‐exome sequencing (WES). Somatic mutations were those detected only in DNA from tumor and absent in DNA from leukocyte. Before library construction, DNA was requantified using a Qubit Fluorometer (Invitrogen) following the manufacture's recommendations. WT‐gene panel libraries were prepared using 10 ng of DNA in the Ion AmpliSeq Library Kit 2.0 (Thermo Fisher Scientific). Whole‐exome sequencing libraries were performed using 1 μg of DNA in the Ion TargetSeq Exome Enrichment kit (Thermo Fisher Scientific).

The libraries were quantified using qPCR with the Ion Library Quantification kit (Thermo Fisher Scientific) before template amplification. Template amplification was performed by emulsion PCR and the enrichment was performed using an Ion PI Template OT2 Kit v3 (Thermo Fisher Scientific). Sequencing was performed using an Ion PI Sequencing 200 Kit v3 with an Ion PI Chip v3 in Ion Proton platform (Thermo Fisher Scientific).

The WT‐gene panel contains ten genes of the microRNA biogenesis pathway (*DROSHA*, *DGCR8*, *RAN*, *XPO5*, *DICER1*, *TARBP2*, *AGO1*, *AGO2*, *GEMIN4*, and *DDX20*), three genes of the Wnt pathway (*APC, CTNNB1,* and *TP53*), and other genes previously reported as being mutated in WT (*WT1*, *WTX*, *DIS3L2*, and *FBXW7*). Whole‐exome sequencing was also performed when somatic mutations identified in genes of the WT‐gene panel were not detected in the patient's body fluids in order to identify additional somatic mutations. The sequencing quality information for WES and WT‐gene panel is shown in Table S2.

### 2.4 Deep amplicon sequencing

For validating the somatic nature of the selected mutations, they were evaluated in 3 ng DNA from tumor and leukocyte matched samples. For that, primers were designed using Primer3 (v. 0.4.0) to generate amplicons of 97‐125 bp in length, embracing the mutations. Polymerase chain reaction was performed using Platinum® Taq DNA Polymerase High Fidelity (Invitrogen). The PCR cycle condition was an initial denaturation at 95°C for 3 minutes; 40 cycles of 95°C 30 seconds, 60°C 30 seconds, and 68°C 30 seconds; and a final extension at 68°C for 10 minutes. For validating more than one somatic mutation detected in one tumor tissue multiplex PCR was performed with a Multiplex PCR Kit (QIAGEN) using the following conditions: initial denaturation at 95°C for 5 minutes; 40 cycles of 94°C 30 seconds, 60°C 45 seconds, and 72°C 30 seconds; and a final extension at 72°C for 10 minutes.

For verifying if PIK3CA‐related overgrowth spectrum (PROS) affected patients of our cohort, *PIK3CA* hotspots (codons 420, 542, 545, 546, 549, and 1047) were evaluated using the same PCR conditions described above.

Validated somatic mutations from tumor tissue were defined as tumor markers and interrogated in all body fluids samples of each patient. Body fluid DNA samples were requantified using a Qubit Fluorometer (Invitrogen) and 3 ng was used as DNA input for the PCR assays. When the samples had not enough DNA for proper Qubit quantification, they were concentrated to about 50% of its initial volume using the CentriVap Concentrator system (Labconco). The PCR products were checked on a 3% agarose gel, followed by library preparation using the Ion Plus Fragment Library Kit (Thermo Fisher Scientific). Library quantification, emulsion PCR, enrichment, and sequencing were performed as mentioned before. Duplicates of body fluid samples positive for somatic mutations were performed when enough DNA from body fluids was available.

### 2.5 Variant calling criteria

For the WT‐gene panel, Ion Reporter 5.2 was used for variant identification, annotation, and selection, with the following criteria: (a) minimum coverage of 400×, (b) minimum variant frequency of 10% in tumor DNA and absent on leukocyte DNA, (c) classified as nonsynonymous variants in coding regions (single nucleotide variants, SNVs; or insertions and deletions, INDELs), (d) not reported in the Single Nucleotide Polymorphism Database (dbSNP) or with a minor allele frequency (MAF) <1%. Selected variants were visually verified using Genomics Workbench software (CLCBio).

For WES, variants of tumor and leukocyte DNA were identified and annotated using the Ion Torrent software variant caller built‐in plugin (v5.0.4.0), and variants detected only in tumor tissue were selected using VarSeq software (v1.4.6) (Golden Helix). The selection criteria were as follows: (a) nonsynonymous variants in coding regions (SNVs or INDELs), (b) not reported in the dbSNP or with MAF < 1%, (c) present in at least 20% of the reads for tumors and missing for leukocytes, (d) quality depth >2, (e) strand bias <0.8, (f) read depth >30×.

For deep amplicon sequencing, BAM files were loaded in Ion Reporter 5.2 and analyzed using custom workflows, with the following criteria: (a) downsample to coverage: 20 000×; (b) VAF ≥ 1% (the criteria was based on negative controls that ranged from 0.00% to 0.33%, as described in Table S3), and (c) variant quality filters was used as default.

## 3 RESULTS

Six female patients (P01‐P06), diagnosed with WT aged between 8 months and 5 years and 11 months, were enrolled in this study. Patients and tumors detailed characteristics are described in Table 1.

**TABLE 1:** Patients and tumors characteristics

	P01	P02	P03	P04	P05	P06
Sex	F	F	F	F	F	F
Age at diagnosis	5 y, 11 mo	3 y, 11 mo	3 y, 5 mo	2 y, 3 mo	8 mo	1 y, 2 mo
Wilms tumor stage (SIOP)	II	IV	V	IV	II	II
Wilms tumor subtype	Regressive	Blastemal	Blastemal	Epithelial	Stromal	Stromal
Risk stratification	Intermediate	High	High	Intermediate	Intermediate	Intermediate
Pretreatment lesion dimensions	130 mm × 124 mm × 103 mm	106 mm × 82 mm	42 mm × 15 mm	170 mm × 153 mm	128 mm × 111 mm	92 mm × 68 mm × 69 mm
Localization of lesion in renal tissue	Middle and upper third of the left kidney	Middle and lower third of the left kidney	Middle and lower third of the left kidney	Middle third of the left kidney, occupying all the left hemiabdomen	All of the right hemiabdomen	All renal parenchyma of the left kidney, with a small posterior cortical remnant
Metastasis	Yes (lungs), relapse 9 mo after WT diagnosis	Yes (lungs), at the time of WT diagnosis	No	Yes (lungs), at the time of WT diagnosis	No	No
Observations	The patient had no clinical evidence for WT progression. In follow up	The patient had no clinical evidence for WT progression. In follow up	Bilateral WT, first treated at another institution. In follow up	Progressed to death	The patient had no clinical evidence for WT progression. In follow up	The patient had no clinical evidence for WT progression. In follow up
Period of follow‐up in this study	1089 d	316 d	1095 d	199 d	294 d	245 d

For patient P01, our aim was to verify if somatic mutations could be detected in plasma and urine of WT patients at diagnosis and, for that, both body fluids samples were collected before treatment. As this patient relapsed with lung metastasis 9 months after WT diagnosis, both tumor DNAs from primary tumor and metastasis were evaluated to identify somatic mutations present in tumor and absent in leukocyte DNA, to be used as personalized tumor markers. No somatic mutations of primary tumor or metastasis DNAs were identified in genes from the WT‐gene panel and, consequently, the DNA samples were submitted to WES for identification of mutations in potential new genes. In the DNA of primary tumor, two somatic mutations were identified: a missense mutation in *INTS1* (c.2257G > A, p.Gly753Ser) with a variant allele fraction (VAF) of 50.00% and a single‐base frameshift deletion in *TNRC18* (c.3499delG, p.Glu1167Argfs*40) with a VAF of 37.29% (Table 2). In the metastasis DNA, four somatic mutations were detected. The two mutations identified in the primary tumor were detected in metastasis with higher VAFs: 85.19% for the *INTS1* (c.2257G > A, p.Gly753Ser) mutation and 87.50% for the *TNRC18* (c.3499delG, p.Glu1167Argfs*40) mutation, compared to 50.00% and 37.29% for the primary tumor, respectively, and two additional missense mutations: *KRT80* (c.932C > T, p.Ser311Phe) with a VAF of 38.80% and *TTI1* (c.1516G > A, p.Asp506Asn) with 38.75% of VAF (Table 2). Deep amplicon sequencing in DNA from both tumors, primary and metastasis, confirmed that *KRT80* and *TTI1* mutations were present only in the metastasis, suggesting that either the primary tumor fragment analyzed was not representative of the entire tumor or that both mutations were a late event of the tumor progression.

**TABLE 2:** Somatic mutations detected in tumors of patients P01‐P06

Patient ID	Tumor	Gene	Mutation type	cDNA change	Protein change	VAF (%)	Detection method	Detection in body fluids before treatment		
								Plasma	Urine sediment	Urine supernatant
P01	Wilms tumor	*INTS1*	Missense	c.2257G > A	p.Gly753Ser	50.00%	WES	No	**YES**	**YES**
		*TNRC18*	Frameshift Deletion	c.3349delG	p.Glu1167Argfs*40	37.29%	WES	No	**YES**	No
	Lung metastasis	*INTS1*	Missense	c.2257G > A	p.Gly753Ser	85.19%	WES	No	**YES**	**YES**
		*TNRC18*	Frameshift Deletion	c.3349delG	p.Glu1167Argfs*40	87.50%	WES	No	**YES**	No
		*KRT80*	Missense	c.932C > T	p.Ser311Phe	38.80%	WES	No	No	No
		*TTI1*	Missense	c.1516G > A	p.Asp506Asn	38.75%	WES	No	No	No
P02	Wilms tumor	ND	—	—	—	—	—	—	—	—
P03	Wilms tumor	*SUPT7L*	Missense	c.1217G > A	p.Arg406His	43.81%	WES	No	No	No
		*KLHL30*	Missense	c.1253C > A	p.Ala418Asp	47.56%	WES	No	No	No
		*EIF4G1*	Missense	c.2843C > T	p.Thr948Ile	39.39%	WES	No	**YES**	**YES**
		*DROSHA*	Missense	c.3665A > G	p.Glu1222Gly	32.00%	WTGP and WES	No	No	No
		*RAD50*	Missense	c.3835C > T	p.Arg1279Cys	29.52%	WES	No	No	No
		*TMED9*	Frameshift Deletion	c.208_209del	p.Asp70Glnfs*47	50.00%	WES	No	No	No
		*MTA2*	Missense	c.187G > T	p.Ala63Ser	53.01%	WES	No	No	No
		*PPP2R1A*	Missense	c.547C > T	p.Arg183Trp	55.00%	WES	No	No	No
P04	Wilms tumor	*SERBP1*	Nonsense	c.1117C > T	p.Arg373*	97.26%	WES	No	No	No
		*WTAP*	Missense	c.485G > A	p.Arg162Gln	98.63%	WES	**YES**	**YES**	**YES**
		*PHF5A*	Missense	c.305G > A	p.Arg102His	97.22%	WES	**YES**	**YES**	**YES**
P05	Wilms tumor	*CTNNB1*	Missense	c.1147T > G	p.Trp383Gly	48.60%	WTGP	**YES**	No	No
P06	Wilms tumor	*CTNNB1*	Missense	c.1149G > T	p.Trp383Cys	47.30%	WTGP	**YES**	—	—
		*WTX*	Nonsense	c.1057C > T	p.Arg353*	45.82%	WTGP	**YES**	—	—

Abbreviation: ND, not detected; WES, whole‐exome sequencing; WTGP, Wilms tumor Gene Panel; VAF, variant allele fraction.

**FIGURE 2:**
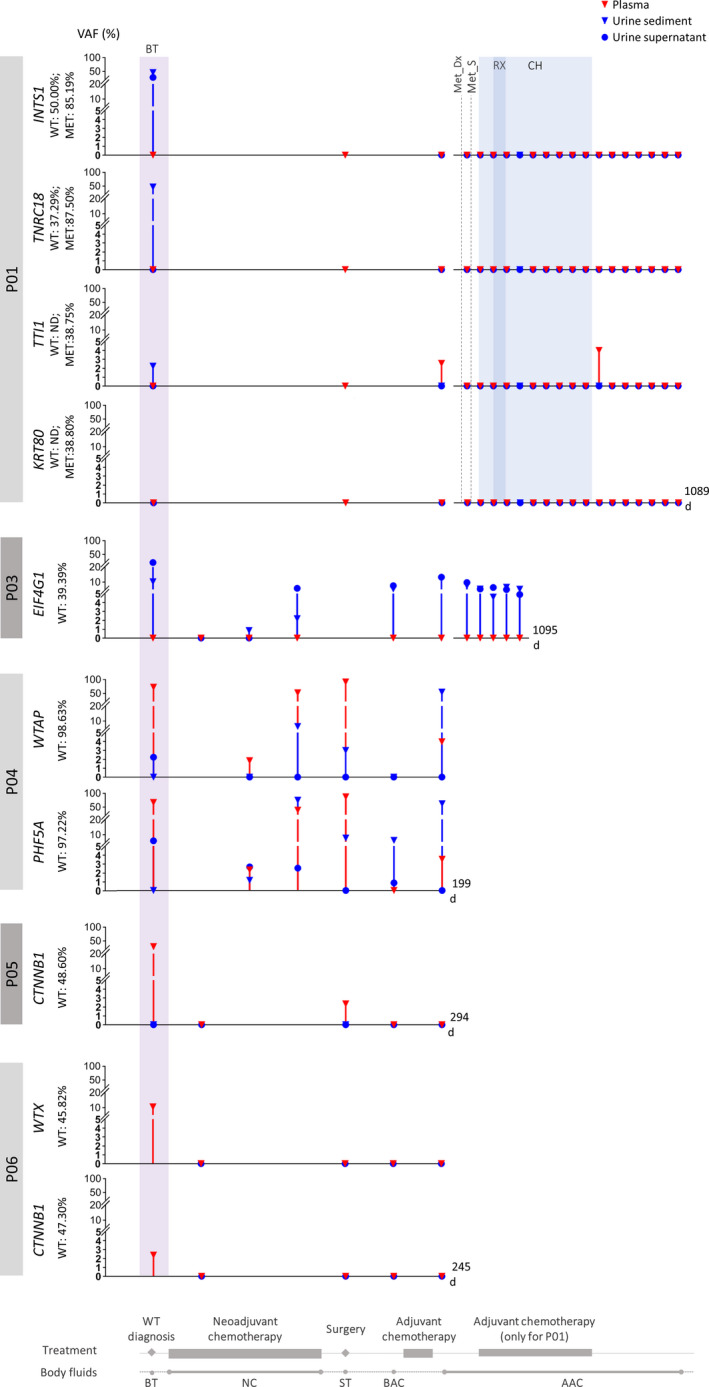
Somatic mutations of patients P01‐P06 in serial body fluids samples during treatment. Patient P01: Met_Dx, metastasis diagnosis; Met_S, metastasis surgery; RX, radiotherapy; CH, chemotherapy; *INTS1* (c.2257G > A, p.Gly753Ser), *TNRC18* (c.3499delG, p.Glu1167Argfs*40), *KRT80* (c.932C > T, p.Ser311Phe), *TTI1* (c.1516G > A, p.Asp506Asn). Patient P03: *EIF4G1* (c.2843C > T, p.Thr948Ile). Patient P04: *WTAP* (c.485G > A, p.Arg162Gln), *PHF5A* (c.305G > A, p.Arg102His). Patient P05: *CTNNB1* (c.1147T > G, p.Trp383Gly). Patient P06: *CTNNB1* (c.1149G > T, p.Trp383Cys), *WTX* (c.1057C > T, p.Arg353*). VAF, variant allele fraction; Treatment, treatment timeline. Body fluids, collection of body fluids samples according to treatment timeline; BT, baseline sample collected before treatment; NC, samples collection during neoadjuvant chemotherapy; ST, sample collected at surgery time; BAC, samples collected before adjuvant chemotherapy; AAC, samples collected after adjuvant chemotherapy

The tracking of somatic mutations in DNA of body fluids was performed before treatment and during adjuvant chemotherapy for both primary and metastatic tumors. The monitoring period for this patient lasted 1089 days. Two somatic mutations were detected before treatment only in urine, in both components, implying the presence of tumor DNA. The *INTS1* (c.2257G > A, p.Gly753Ser) mutation was detected in urine sediment with a VAF of 46% and the urine supernatant with 28%. The *TNRC18* (c.3499delG, p.Glu1167Argfs*40) mutation was present only in the urine sediment with a VAF of 47%. Interestingly, the *TTI1* (c.1516G > A, p.Asp506Asn) mutation detected only in lung metastasis was spotted in the DNA of the baseline urine sediment sample with a VAF of 2.2% (Figure 2). In plasma DNA, this mutation was detected during monitoring period only in 2 out of 19 samples. Interestingly, in plasma DNA, the first detection of the *TTI1* mutation was in a sample collected after adjuvant chemotherapy for treating primary tumor, and the second was after chemotherapy for treating lung metastasis, with VAFs of 2.6% and 4.2%, respectively. The imaging exams and clinical parameters of this patient showed no signal of disease progression. The *KRT80* (c.932C > T, p.Ser311Phe) mutation, found in the metastasis with a VAF of 38.80%, was not detected in the baseline or any subsequent body fluid sample (Figure 2; Table S4).

Five additional WT patients (P02‐P06) were included in this study (Table 1) to investigate the possibility of tracking somatic mutations in the DNA of body fluids to monitor response to neoadjuvant chemotherapy as well as adjuvant chemotherapy response and disease progression. Thus, for these patients, body fluids were collected during neoadjuvant chemotherapy exposure as presented in Figure 1.

First, we sequenced both matched tumor tissue and leukocyte DNAs using the WT‐gene panel and were able to uncover somatic mutations in the tumors of three patients (P03, P05, and P06; Table 2). The tumor from P03 presented a mutation in *DROSHA* (c.3665A > G, p.Glu1222Gly) with a VAF of 32%. The tumor from P05 harbored a mutation in *CTNNB1* (c.1147T > G, p.Trp383Gly) with 48.6% of VAF, and tumor from P06 presented two mutations: one in *CTNNB1* (c.1149G > T, p.Trp383Cys, affecting the same codon of tumor from P05), and one in *WTX* (c.1057C > T, p.Arg353*) with VAFs of 47.30% and 45.82%, respectively.

For the patients in whom no specific tumor mutation was detected in genes of the panel (P02 and P04), WES was performed (Table 2). Three somatic mutations were detected in the tumor of P04: one nonsense mutation in *SERBP1* (c.1117C > T, p.Arg373*) with 97.26% of VAF, and missense mutations in *WTAP* (c.485G > A, p.Arg162Gln) and *PHF5A* (c.305G > A, p.Arg102His) with VAFs of 98.63% and 97.22%, respectively. Surprisingly, no somatic mutation was identified in P02; therefore, this patient's body fluids could not be analyzed.

The presence of tumor DNA in body fluids of patients P03‐P06, in whom a somatic mutation in tumor was identified, was assessed in body fluid samples collected before treatment (baseline). DNA from baseline samples of urine components and plasma were analyzed for P03, P04, and P05. For patient P06, only plasma was collected before treatment. Somatic mutations were detected in at least one baseline body fluid in three of them (P04, P05, and P06). In P04, two of the three mutations, *WTAP* (c.485G > A, p.Arg162Gln) and *PHF5A* (c.305G > A, p.Arg102His), were identified in DNA from plasma with VAFs of 71.37% and 68.74%, respectively, and in DNA from urine sediment with VAFs of 2.24% and 5.79%, respectively. In P05, the mutation in *CTNNB1* (c.1147T > G, p.Trp383Gly) was detected only in plasma with 29.17% of VAF. In P06, the mutations in *CTNNB1* (c.1149G > T, p.Trp383Cys) and *WTX* (c.1057C > T, p.Arg353*) were identified in plasma with VAFs of 2.75% and 10.58%, respectively. However, as only plasma was collected at this time point for this patient, we were unable to verify if these mutations were present in the urine components before treatment. Finally, in P03, the mutation in *DROSHA* (c.3665A > G, p.Glu1222Gly), detected in tumor with VAF of 32.00%, was not identified in the baseline samples. Thus, for this patient WES was performed in tumor and leukocyte DNAs for detecting additional somatic mutations. Seven additional somatic mutations were identified: missense mutations in *SUPT7L* (c.1217G > A, p.Arg406His), *KLHL30* (c.1253C > A, p.Ala418Asp), *EIF4G1* (c.2843C > T, p.Thr948Ile), *RAD50* (c.3835C > T, p.Arg1279Cys), *MTA2* (c.187G > T, p.Ala63Ser), and *PPP2R1A* (c.547C > T, p.Arg183Trp) with VAFs of 43.81%, 47.56%, 39.39%, 29.52%, 53.01%, and 55.00%, respectively, and one frameshift INDEL in *TMED9* (c.208_212delInsTTG, p.Asp70Glnfs*47) with 50% of VAF. Intriguingly, only the tumor mutation in *EIF4G1* was detected in the sediment and the supernatant of the baseline urine sample. Somatic mutations identified in the baseline DNAs of plasma and of the two components of urine of all patients are presented in Figure 2 and Table S4.

Next, for monitoring chemotherapy response, the somatic mutations were tracked in the DNAs of body fluid samples collected during neoadjuvant chemotherapy, surgery, and adjuvant chemotherapy. For these patients, a decrease in the VAF of somatic mutations was observed after the initiation of neoadjuvant chemotherapy, suggesting the effect of chemotherapy on the tumor. However, for two patients (P03 and P04), the presence of somatic mutations became persistently detectable in serial body fluid samples at the end of neoadjuvant and during adjuvant treatments (Figure 2; Table S4).

For P03, who had a partial nephrectomy, we evaluated three additional body fluid collections (AAC2, AAC3, and AAC4) and the *EIF4G1* mutation was still detectable in the urine components with a VAF of 6.32%, 3.59%, and 4.24% for the urine sediment and of 9.9%, 4.76%, and 5.7% for the urine supernatant, respectively. Neither clinical manifestation nor imaging exam supports the presence of residual disease or WT progression in this patient. This patient is still under clinical follow‐up.

For P04, the two somatic mutations in *WTAP* and *PHF5A* genes were detected in nearly all body fluid collections at a higher VAF than the mutations observed in body fluid samples of other patients. This patient later progressed to death by cardiorespiratory arrest after pleural invasion of the lung metastasis.

For P05, during monitoring we detected the *CTNNB1* mutation at the time of surgery (ST) with a VAF of 2.35%. This mutation was not detected in the two body fluid samples collected postoperatively (BAC and AAC1). This patient had no clinical evidence for WT progression.

For P06, in subsequent body fluid samples collected during monitoring period, the somatic mutations could not be detected. This patient also had no clinical evidence for WT progression.

## 4 DISCUSSION

Liquid biopsy (LB) for tracking somatic mutations in plasma has been recognized as a plausible alternative in oncology for monitoring treatment response and disease progression. The use of plasma as a LB has been widely explored in clinical practice showing a huge potential for capturing alterations in the mutational architecture of a solid tumor by the capability of tumor DNA being serially sampled in a minimally invasive manner. Besides plasma, urine has also been usually mentioned as a tool of huge application in genitourinary cancer. Urine can be obtained in a noninvasive way, making them perfectly suitable for analysis of children with renal cancer, such as WT. Thus, in the current study, we evaluated the utility of somatic mutations assessment as tumor markers in body fluids, aiming to contribute with complementary tools for monitoring treatment response of WT patients.

Wilms tumor is considered a rare disease representing 95% of all malignant renal tumors in pediatric patients. In this study, we were able to track the mutations exclusively detected in DNA of tumors in plasma and urine of five WT patients, in whom a minimum of one tumor somatic mutation was identified. Our results showed that, in at least one body fluid analyzed, somatic mutations were detected allowing the patient monitoring during treatment. Additionally, we observed that neither plasma nor urine alone were enough to detect somatic mutations for all WT patients. In other words, only by interrogating DNA from both body fluids we were able to track tumor mutations during treatment to be correlated with clinical information. Regarding both components of urine, neither the sediment nor the supernatant stood out for the uncovering of tumor DNA.

In terms of the capability to track somatic mutations in body fluids of WT patients, other studies presented promising results. Treger and colleagues^29^ have checked by Droplet digital polymerase chain reaction (ddPCR) tumor somatic *TP53* mutations in serum, plasma, and urine samples taken preoperatively from four DAWT cases and were able to detect mutations in the serum and/or plasma of all patients evaluated, but not in all urine samples. Additionally, Jiménez and colleagues^30^ used WES in DNA from plasma and their matched tumor and leukocyte samples of patients with pediatric renal tumors for detecting tumor specific alterations (single nucleotide variants and copy number variations) and were able to detect somatic mutations in plasma in 85.7% of 14 WT patients analyzed. Our study, in concordance with Jiménez and colleagues, also showed that not all mutations identified in the analyzed tumor fragment were detected in body fluids, supporting that the biology of tumor DNA shed in body fluids is a complex mechanism that needs further investigation.

The shedding of circulating tumor DNA is still a matter of investigation, as it may depend on several aspects. Plasma usually presents DNA from cell apoptosis, necrosis, and from active release and is typically fragmented.^31^, ^32^ The amount of tumor DNA in circulation also depend on the tumor characteristics, such as mitotic rate, vascularization, necrosis, among others,^31^, ^33^ and also cancer staging. As for urine, its use for clinical applications and the biology of tumor DNA in both urine components present controversies and are in constant debate. In principle, the urine sediment is composed of exfoliated cells from the genitourinary tract and its mainly intact DNA^18^, ^34^; and the urine supernatant contains highly degraded DNA from the circulation after cell apoptosis and glomerular filtration.^35^ Some authors argue that most of the tumor DNA in the urine from patients with urogenital cancer is the result of direct transport of tumor cells or their degradation products into the urine,^36^ suggesting that the urine sediment is the best component for its detection. In contrast, another study has shown that in urothelial bladder cancer, the DNA of the urine supernatant allowed a better analysis of the tumor profile as it had a higher tumor DNA load than the urine sediment.^25^

Tumor cells might have different levels of sensitivity to treatment resulting in apoptosis or necrosis of cells from specific clone subpopulations,^37^ consequently changing the mutational architecture of solid tumors and probably acting in the different ability to shed DNA in body fluids. These aspects could at least partially explain the differences in the profile of somatic variants detected in body fluids samples when compared to the profile detected in the corresponding tumor tissue DNA, and also in the difference in the VAF detected in different time points during the monitoring period. These aspects can explain the results obtained for patient P03, who had only the *EIF4G1* mutation detected in urine components (one out of eight somatic mutations identified in her tumor tissue DNA); or for patient P04, who had all three somatic mutations (*SERBP1*, *WTAP,* and *PHF5A*) in very high VAFs in tumor tissue (97.26%, 98.63%, and 97.22%, respectively—Table 2) and only two of them (*WTAP* and *PHF5A*) detected in DNA of body fluids before and during patient monitoring (Table S4; Figure 2).

This current study also confirmed that the WT‐associated panel containing genes frequently mutated in WT, previously used by us,^12^ has about 50% of sensitivity for detecting somatic mutations in WT and that the strategy of evaluating the whole exome of tumor samples negative for somatic mutations in genes of the panel is quite necessary for identifying markers to be screened in DNA from body fluids.

In terms of intratumor heterogeneity, along with other studies,^38^, ^39^ our results also showed that liquid biopsy might be able to identify somatic mutations missed in the analysis of fragments of tumor tissue and that one fragment of WT tissue might not completely represent the entire tumor. In the patient P01, the *TTI1* (c.1516G > A, p.Asp506Asn) mutation was detected in the urine sediment, that represents the urogenital cells released directly into the urine, at the time of diagnosis. The fact that this mutation was not detected in the WT fragment obtained from the surgical specimen strongly suggests that, for this specific case, the urine sediment was more representative of the entire tumor. Still on the *TTI1* mutation, it was later detected in the plasma of patient P01 in two moments shortly after the adjuvant treatments. Interestingly, *TTI1* is a DNA damage response regulator involved in cell resistance to stresses such as ionizing radiation, ultraviolet light, and mitomycin C. Mitomycin C is an antineoplastic antibiotic that binds to DNA, causing disruption to the duplication and transcription processes.^40^ Its mechanism of action is similar to those of actinomycin D^41^ and doxorubicin,^42^ the chemotherapeutic agents used by P01 in the adjuvant treatments for WT and the lung metastasis. The clinical value of the detection of the *TTI1* mutations in isolated points of treatment is not clear.

Regarding patient P03, in spite of the fact that the detection in urine sediment of traces of tumor DNA have persisted at similar VAF, the patient has neither clinical manifestation nor imaging exams that support the presence of active tumor. Thus, patient monitoring by LB continues being performed in close contact with clinicians to support treatment adjustments if the amount of tumor DNA increases in the urine sediment or in other body fluids. In contrast, we persistently detected a high VAF of somatic mutations in the DNA of serial body fluid samples collected from P04, who progressed to death.

Recently, new and exciting findings came in the literature showing that WT is part of the lesion range of *PIK3CA* related overgrowth spectrum (PROS),^43^ which refers to a group of syndromes characterized by malformation and tissue overgrowth. Patients with these conditions harbor *PIK3CA* somatic mutations in preneoplastic and nontumorous cells, not necessarily being detected in their leukocytes DNA.^44^, ^45^ In this study we investigated, by deep amplicon sequencing with high coverage which is able to detect low frequency mutations, six *PIK3CA* activating hotspot mutations in codons 420, 542, 545, 546, 549, and 1047 in both DNAs from WT and leukocyte of all patients. No mutation was detected neither in DNA from WT nor in DNA from leukocyte in any of the patients. Thus, we can just state that the participating patients of this study probably do not have PROS, which could cause the presence of somatic mutations in other body tissues and interfere in the liquid biopsy results.

Altogether, this study is the first one to evaluate, in a personalized format, somatic mutations in serial samples of urine and plasma obtained before, during, and after treatment. Thus, even with limitations such as the small sample size, our study demonstrated a concordance of liquid biopsy results and clinical aspects of WT patients, reinforced by patient P04 persistent high VAF of somatic mutations in body fluids and her unfavorable clinical outcome. Larger studies are required to assess the clinical utility of performing LB on WT patients regarding the choice of optimal therapies and monitoring treatment response.

## 5 CONCLUSIONS

In this study, through the combined evaluation of urine and plasma samples, we were able to detect at least one somatic mutation in at least one of the body fluids in all WT patients that were analyzed, demonstrating the potential use of urine and plasma as a minimally invasive strategy for monitoring treatment response for WT patients.

## COMPETING INTERESTS

The authors declare that they have no competing interests.

## AUTHORS' CONTRIBUTIONS

The study was conceived by ACKM and DMC. Patients were recruited by CMLC. Pathology analysis was performed by IWC. Tumor size and localization were obtained by TC scan by PB. Surgeries were performed and tumors were collected by MLP. Experiments were performed by ACKM and BDFB. Bioinformatic and data interpretation analysis were performed by ACKM, BDFB, DMC, JESS, and SJS. The manuscript was written by ACKM and DMC and edited by SJS, BDFB, and JESS. All authors are in accordance with the final version of the manuscript.

## ETHICS APPROVAL AND CONSENT TO PARTICIPATE

This study was approved by the Ethics Committee of A. C. Camargo Cancer Center under the number 2222/16. Informed consent was obtained from the parents or legal guardians of all participants included in this study.

## Supporting information

Table S1

Table S2

Table S3

Table S4

## Data Availability

The data that support the findings of this study are available from the corresponding author upon reasonable request.
